# A randomized, double-blind study of repeated incobotulinumtoxinA (Xeomin^®^) in cervical dystonia

**DOI:** 10.1007/s00702-013-1048-3

**Published:** 2013-06-19

**Authors:** Virgilio Gerald H. Evidente, Hubert H. Fernandez, Mark S. LeDoux, Allison Brashear, Susanne Grafe, Angelika Hanschmann, Cynthia L. Comella

**Affiliations:** 1 Movement Disorders Center of Arizona, 9590 E. Ironwood Square Drive, #225, Scottsdale, AZ 85258 USA; 2 Cleveland Clinic, Cleveland, OH USA; 3 University of Tennessee Health Science Center, Memphis, TN USA; 4 Wake Forest School of Medicine, Winston-Salem, NC USA; 5 Merz Pharmaceuticals GmbH, Frankfurt, Germany; 6 Rush University Medical Center, Chicago, IL USA

**Keywords:** Dystonia, Xeomin, IncobotulinumtoxinA, NT 201, Botulinum toxin

## Abstract

IncobotulinumtoxinA (Xeomin^®^, NT 201), a preparation without accessory (complexing) proteins, has shown comparable efficacy and safety to onabotulinumtoxinA in treating cervical dystonia (CD). This study evaluated the efficacy and safety of repeated incobotulinumtoxinA injections in subjects with CD. Following a ≤20-week placebo-controlled, randomized, double-blind, single-dose main period, subjects could enter a ≤68-week prospective, randomized, double-blind, repeated-dose, flexible-interval (minimum 6 weeks) extension period with 240 U or 120 U of incobotulinumtoxinA (≤5 injections). Outcome measures included the Toronto Western Spasmodic Torticollis Rating Scale (TWSTRS) and adverse events (AEs). Of 219 subjects completing the main period, 214 were randomized in the extension period to receive incobotulinumtoxinA 240 U (*n* = 111) or 120 U (*n* = 103); 169 subjects completed the extension period, with 90 receiving five injection sessions. Both doses of incobotulinumtoxinA provided statistically significant and clinically relevant improvements in mean TWSTRS-Total, -Severity, -Disability, and -Pain scores, from each injection session to respective 4-week follow-up visits. The most frequently reported AE was dysphagia (240 U: 23.4 %; 120 U: 12.6 %), which did not result in any study withdrawals. There was no impact of injection interval on the incidence of AEs (post hoc analysis). A major limitation of this study was the fixed dose design requested by regulatory authorities, which does not reflect clinical practice. However, repeated incobotulinumtoxinA injections (240 or 120 U; flexible intervals) provided sustained efficacy and were well tolerated, with no unexpected safety risks following repeated injections. The incidence of AEs was similar in subjects requiring repeated injections at shorter intervals (≤12 weeks) compared with those treated using longer intervals (>12 weeks).

## Introduction

Cervical dystonia (CD) is a focal dystonia characterized by involuntary activation of the muscles of the neck and shoulders, resulting in abnormal repetitive and/or sustained posturing. Pain is a recognized clinical feature of CD: over two-thirds of subjects have associated neck pain (Chan et al. 1991). The efficacy and tolerability of botulinum toxin for the treatment of CD are supported by Class I evidence (Simpson et al. 2008).

When isolated from *Clostridium botulinum* cultures, botulinum toxin is composed of a 150-kDa core neurotoxin and accessory (complexing) proteins, including hemagglutinin and non-toxin non-hemagglutinin (NTNH) proteins (Inoue et al. 1996). IncobotulinumtoxinA (Xeomin^®^; also known as NT 201; Merz Pharmaceuticals GmbH, Frankfurt, Germany) is a purified form of botulinum toxin type A that contains no accessory proteins and has a high specific biological activity (Frevert 2009, 2010; Frevert and Dressler, 2010, Dressler 2012). IncobotulinumtoxinA has shown comparable efficacy and safety to onabotulinumtoxinA in the treatment of CD when used in a 1:1 dosing ratio (Benecke et al. 2005) and was equipotent to onabotulinumtoxinA in a mouse toxicity (LD_50_) assay (Dressler et al. 2011).

In the double-blind, randomized, placebo-controlled main period (MP) of a phase 3 study, incobotulinumtoxinA (240 or 120 U) significantly improved Toronto Western Spasmodic Torticollis Rating Scale (TWSTRS)-Total scores and subscores compared with placebo in subjects with primary CD of a predominantly rotational form (Comella et al. 2011). While this demonstrates the efficacy of a single set of incobotulinumtoxinA injections, the majority of subjects with focal dystonia require long-term treatment. Herein, we report data from the double-blind extension period (EP) of this study, which investigated the safety and efficacy of repeated injection sessions of incobotulinumtoxinA (240 or 120 U) in subjects with CD over a 68-week period (including a 20-week safety period). This is the first randomized trial in CD where the design of the EP included flexible injection intervals (minimum 6 weeks) to allow tailoring of treatment to meet individual subject needs.

## Methods

The design and results of the placebo-controlled MP of this double-blind, randomized, parallel-group phase three trial (clinicaltrials.gov identification number: NCT00407030; MRZ 60201–0408) have been reported (Comella et al. 2011). The EP had a randomized, double-blind, parallel-group design and was conducted at 37 centers across the USA. The total duration of this incobotulinumtoxinA long-term study (MP plus EP) was up to 88 weeks. The respective Institutional Review Boards approved the study protocol and the informed consent process. The study was conducted in accordance with the ethical principles outlined in the declaration of Helsinki and was consistent with good clinical practice and the applicable regulatory requirements. Both the MP and EP were monitored by an independent Data Safety Monitoring Board (DSMB).

### Subjects

In the MP, pre-treated and treatment-naïve subjects were eligible for enrollment (Comella et al. 2011).

Subjects could enter the EP if they had completed the last visit of the MP and had a need for re-injection, which the investigator agreed was necessary, as defined by a TWSTRS-Total score ≥20 points.

### Randomization

Before enrollment into the MP, subjects were randomized to receive incobotulinumtoxinA (240 or 120 U) or placebo in the MP. At this time point, independently from randomization to MP treatment, subjects were also randomized to receive incobotulinumtoxinA injections at total doses of either 240 or 120 U in the EP. Randomization was performed using RANCODE version 3.6 (IDV, Gauting, Germany). Block-wise randomization (block size of six) ensured a balanced treatment assignment for each center. Subjects, investigators, medical staff, and all staff at the sponsoring company and clinical research organization were blinded to treatment.

### Treatment and follow-up visits

In the EP, subjects received incobotulinumtoxinA at total doses of 240 or 120 U in an equal total volume of 4.8 mL. Muscles treated, number of injection sites per muscle, use of electromyographic guidance, and doses injected at each site were at the discretion of the investigator. All subjects could receive one to five injection sessions of incobotulinumtoxinA as clinically required over the 48 weeks. The interval between injection sessions was flexible, with a minimum interval of 6 weeks up to the time when subjects expressed a need for a re-injection. To be re-injected, subjects had to spontaneously request re-treatment and have a TWSTRS-Total score of ≥20.

**Fig. 1:**
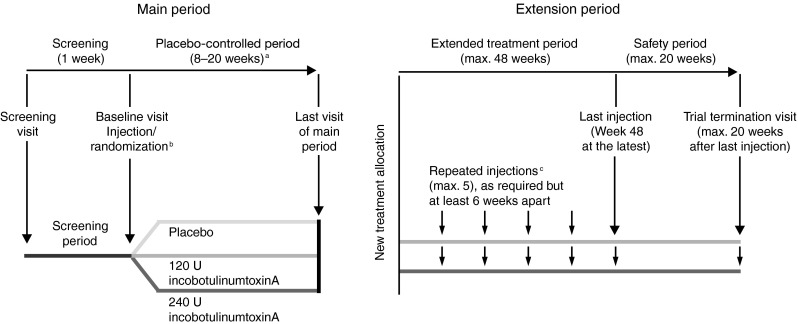
Study design. The maximum duration of the extension period for each individual subject was 68 weeks. ^a^Individual duration of placebo-controlled period per subject. ^b^Telephone contact 1 week after injection session, follow-up visits 4 and 8 weeks after injection session. ^c^Telephone contacts 1 week after each injection session, follow-up visits 4 weeks after each injection session

A mandatory telephone call was made on Day 7 ± 1 and a follow-up visit took place on Day 28 ± 3 after each injection session. The Trial Termination Visit (TTV) was performed 20 weeks (± 3 days) after the last injection session or when the subject expressed a need for a new injection of botulinum toxin other than study medication, whichever came first (Fig. 1).

### Efficacy assessments

The primary and secondary efficacy variables for the MP have been reported previously (Comella et al. 2011). In the EP, the following efficacy variables were assessed: TWSTRS-Total and subscale scores (Disability, Severity, and Pain) (Consky and Lang 1994), Investigator Global Assessment of Efficacy (IGAE), and Patient Evaluation of Global Response (PEGR) (adapted from Wissel et al. (2000). TWSTRS-Total and subscale scores were assessed at each injection session, each follow-up visit, and the TTV; analyses included changes from each injection session to the follow-up visits, and changes from the first injection session in the EP to the TTV. Assessments were only performed by investigators who had been appropriately trained, and the same investigator performed all assessments for a given subject. The IGAE consists of a 4-point Likert scale ranging from 1 (very good) to 4 (poor), and the PEGR scale ranges from –4 (very marked worsening) to +4 (complete abolishment of all signs and symptoms). IGAE and PEGR were recorded at all injection visits of the EP (except the first) and the TTV, for the assessment of the respective previous injection cycle.

### Safety assessments

Subjects were requested to report all adverse events (AEs) to the investigator. At each visit and telephone contact, AEs were evaluated and subjects were specifically asked to report any swallowing difficulties using a 5-point dysphagia rating scale as 0 (absent, no swallowing difficulties), 1 (mild swallowing difficulties), 2 [moderate, when swallowing solid meals (e.g. meat)], 3 (severe, with swallowing difficulties and requiring a change in diet), or 4 (swallowing not possible, resulting in weight loss) (Comella et al. 1992). Any increase in the dysphagia scale score after an injection session was classified as an AE.

Vital signs, including systolic/diastolic blood pressure, respiratory rate, and heart rate, were assessed at each visit. Physical and neurological examinations were performed at the third injection visit of the EP and the TTV. Blood samples for clinical chemistry and hematology, and for the determination of antibodies against botulinum toxin type A, were taken at all visits for evaluation at a central laboratory. Antibodies against botulinum neurotoxin type A were assessed by a validated fluorescence immunoassay for antibodies (FIA-AB). To identify neutralizing antibodies, samples that were positive in the FIA-AB were tested with a mouse ex vivo hemidiaphragm assay (HDA) (Göschel et al.^1997^; Sesardic et al. 2004). FIA-ABs were performed by BioProof AG, Munich, Germany, and HDAs were performed by Toxogen GmbH, Hannover, Germany. For the assessment of the respective previous injection cycle, the Investigator Global Assessment of Tolerability (IGAT) scale, a 4-point Likert scale ranging from 1 (very good) to 4 (poor) was performed at all injection visits of the EP (except the first) and the TTV.

### Statistical methodology

Efficacy results represent the intent-to-treat population, and safety data represent the evaluable-for-safety population (both defined as all subjects who received any amount of study medication during the MP or EP). Study completers (as defined at a blind data review meeting during the EP and prior to data availability) were required to have met one of the following criteria: subjects who completed the trial according to the investigator; subjects who did not have a TTV, but participated in the EP for ≥48 weeks; subjects who received onabotulinumtoxinA but participated in the EP for at least 48 weeks prior to onabotulinumtoxinA injection; or subjects who received the maximum of five injection sessions, even though the minimum EP duration of 48 weeks was not fulfilled.

Statistical analyses were performed with the SAS™ System (Cary, NC, USA), version 8.2 or later. TWSTRS scores were analyzed using exploratory t-tests.

AEs were encoded using the medical dictionary for regulatory activities, version 9.1. In a post hoc analysis, a Chi-square test was used to compare the overall occurrence of AEs between groups of subjects with different median injection intervals (6 to ≤10 weeks, >10 to ≤12 weeks, >12 to ≤14 weeks, or >14 weeks).

## Results

### Subjects

**Fig. 2:**
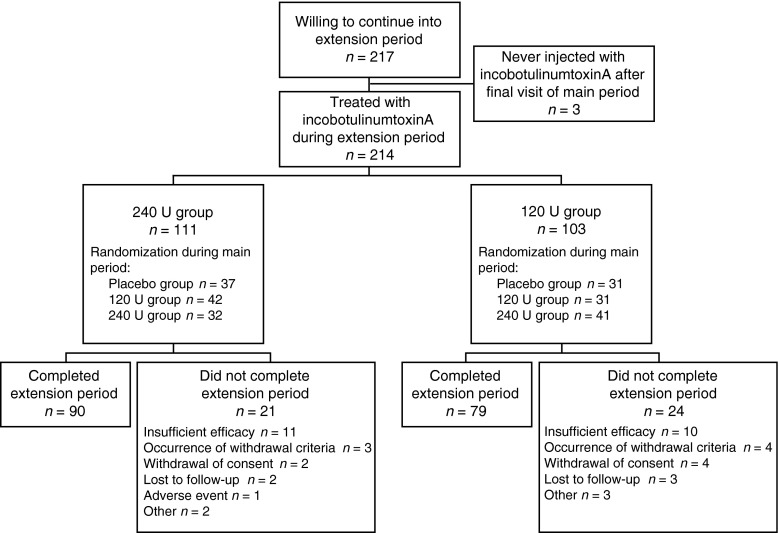
Subject flow diagram

Of the 219 subjects who completed the MP, 217 enrolled in the EP. Of those enrolled, three subjects showed no need for re-injection at 20 weeks after the MP injection session, while 214 subjects who received either incobotulinumtoxinA (240 or 120 U) or placebo in the MP were randomized to receive incobotulinumtoxinA 240 U (*n* = 111) or 120 U (*n* = 103; Fig. 2). In total, 169 subjects (79.0 %) completed the EP [240 U, *n* = 90 (81 %); 120 U, *n* = 79 (77 %)] (Fig. 2), with 90 subjects receiving the maximum of five injection sessions (240 U, *n* = 53; 120 U, *n* = 37). The first subject entered the EP on October 5, 2006, and the last subject completed the study on June 19, 2009.

Demographic characteristics and TWSTRS-Total scores and subscores were well balanced between the dose groups at EP baseline (Table 1). Prior to the MP, 132 had been previously treated with botulinum toxin, and 82 had been treatment-naïve. Swallowing difficulties, as recorded using the dysphagia scale, were reported by 14.6 % of subjects prior to the MP baseline. Demographic data for pre-treated and treatment-naïve subjects were comparable with the total population data of the respective dose groups.

**Table 1:** Characteristics of subject population at EP baseline

	240 U group (*n* = 111)	120 U group (*n* = 103)
Male, *n* (%)	42 (37.8)	34 (33.0)
Mean age, years (SD)	52.4 (12.0)	53.6 (11.2)
Mean BMI, kg/m^2^ (SD)	26.6 (4.9)	27.6 (5.5)
Race, *n* (%)		
American Indian or Alaskan Native	0	1 (1.0)
Asian	1 (0.9)	2 (1.9)
Black or African American	3 (2.7)	1 (1.0)
Hispanic or Latino	4 (3.6)	6 (5.8)
White	103 (92.8)	93 (90.3)
Treatment in MP, *n* (%)		
IncobotulinumtoxinA 240 U	32 (28.8)	41 (39.8)
IncobotulinumtoxinA 120 U	42 (37.8)	31 (30.1)
Placebo	37 (33.3)	31 (30.1)
Mean TWSTRS-Total score (SD)	40.2 (9.6)	41.1 (10.6)
Mean TWSTRS-Severity score (SD)	17.5 (4.4)	17.8 (4.5)
Mean TWSTRS-Disability score (SD)	12.1 (4.4)	12.2 (5.1)
Mean TWSTRS-Pain score (SD)	10.5 (4.1)	11.1 (4.1)

*BMI* body mass index, *MP* main period, *SD* standard deviation, *TWSTRS* Toronto Western Spasmodic Torticollis Rating Scale

### Treatment efficacy

**Fig. 3:**
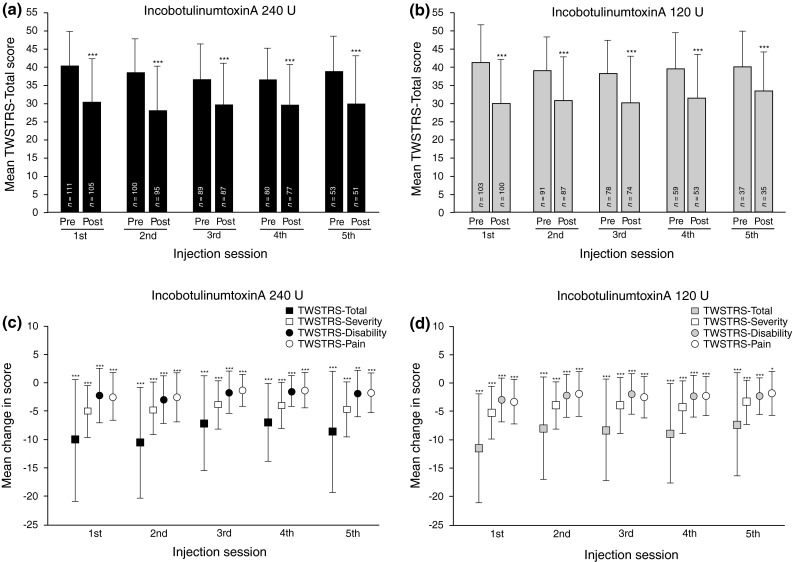
TWSTRS-Total and subscores at each injection session of the EP and the respective follow-up visits 4 weeks later (ITT population). Mean TWSTRS-Total scores in 240 U dose group (**a**); mean TWSTRS-Total scores in 120 U dose group (**b**); mean changes in TWSTRS-Total scores and subscores in 240 U dose group (**c**); mean changes in TWSTRS-Total scores and subscores in 120 U dose group (**d**) *p < 0.05; **p < 0.01; ***p < 0.001*. Error bars* represent standard deviation Numbers of subjects as shown in Fig. 3a and b, with the following exceptions: Injection session 1, 120 U: TWSTRS-Severity and -Disability subscales, *n* = 101 Injection session 3, 240 U: TWSTRS-Pain subscale, *n* = 86 Injection session 3, 120 U: TWSTRS-Severity, -Disability, and -Pain subscales, *n* = 73 *EP* extension period, *ITT* intent-to-treat, Pre at each injection session, *Post* at a follow-up visit 4 weeks after the respective injection session, *TWSTRS* Toronto Western Spasmodic Torticollis Rating Scale

Both doses of incobotulinumtoxinA significantly improved mean TWSTRS-Total score at 4 weeks after each injection session throughout the EP (*p* < 0.001 vs injection visit; Fig. 3a, b). There were also significant mean improvements [standard deviation (SD)] in TWSTRS-Total scores between the first injection session of the EP and the TTV, in both dose groups [240 U (*n* = 81), –4.5 (7.82); 120 U (*n* = 66), −6.7 (9.20); *p* < 0.001 for both groups]. Mean TWSTRS-Severity, TWSTRS-Disability, and TWSTRS-Pain subscales followed a pattern similar to the respective mean TWSTRS-Total scores (Fig. 3c, d). There were significant mean changes from all injection sessions to their respective 4-week follow-up visits in the 240 and 120 U groups for all subscale scores (*p* ≤ 0.016). Treatment differences (mean changes in TWSTRS-Total score and mean changes in TWSTRS subscale scores) between the 240 and 120 U groups were statistically not significant.

Treatment efficacy was assessed by the investigator as “very good” or “good” in the majority of subjects for injection cycles 1–4 and at the TTV [240 U: 75/100 (75.0 %), 77/89 (86.5 %), 64/80 (80.0 %), 43/53 (81.1 %), 73/101 (72.3 %); 120 U: 64/91 (70.3 %), 59/79 (74.7 %), 43/59 (72.9 %), 27/37 (73.0 %), 71/91 (76.3 %), respectively]. Mean scores remained similar throughout the EP (240 U, 1.7–2.0 points; 120 U, 1.8–2.0 points).

At least a moderate improvement (≥2 points) in Patient Evaluation of Global Response (PEGR) was reported by the majority of subjects for injection cycles 1–4 and at the TTV [240 U: 70/100 (70.0 %), 70/89 (78.7 %), 60/80 (75.0 %), 35/53 (66.0 %), 63/101 (62.4 %); 120 U: 59/91 (64.8 %), 51/79 (64.6 %), 36/59 (61.0 %), 23/37 (62.2 %), 58/90 (64.4 %), respectively].

### Investigator global assessment of tolerability (IGAT)

IGAT ratings were “very good” or “good” for the majority of subjects for injection cycles 1–4 and at the TTV [240 U group: 94/100 (94.0 %), 88/89 (98.9 %), 77/80 (96.3 %), 52/53 (98.1 %), 94/102 (92.2 %); 120 U group: 87/91 (95.6 %), 77/79 (97.5 %), 54/59 (91.5 %), 37/37 (100.0 %), 87/93 (93.5 %), respectively].

### Adverse events

During the 68-week EP (≤5 injection sessions), the incidence of AEs per injection interval was 38.8–61.3 % in the 240 U group and 29.7–47.6 % in the 120 U group, while the incidence of adverse drug reactions (ADRs) was 5.4–20.4 % in the 240 U group and 10.0–28.8 % in the 120 U group. The overall incidences of ADRs for all injection intervals are shown in Table 2. Dysphagia, assessed using a 5-point dysphagia scale, was the most frequently reported ADR, with a greater incidence per injection interval in the 240 U group (ranging from 3.8 to 13.5 %) than the 120 U group (ranging from 1.3 to 5.8 %). No subjects withdrew from the study due to dysphagia.

**Table 2:** ADRs affecting ≥5 % of subjects over all injection intervals (evaluable-for-safety population)

ADR, *n* (%)	240 U group (*n* = 111)	120 U group (*n* = 103)
Any ADR	48 (43.2)	38 (36.9)
Dysphagia^a^	26 (23.4)	11 (10.7)
Injection-site pain	13 (11.7)	7 (6.8)
Muscular weakness	7 (6.3)	8 (7.8)
Neck pain	8 (7.2)	7 (6.8)
Musculoskeletal pain	6 (5.4)	5 (4.9)

*ADR* adverse drug reaction

^a^Subjects were specifically asked to report any swallowing difficulties using a 5-point dysphagia scale (Comella et al. 1992); increase in score after an injection session was considered as an adverse event

Most ADRs were of mild (*n* = 58, 27.1 %) or moderate (*n* = 38, 17.8 %) intensity. Severe ADRs were reported by seven subjects (6.3 %) in the 240 U group and eight (7.8 %) in the 120 U group. The most common severe ADRs were neck pain (*n* = 5, 2.3 %), musculoskeletal pain (*n* = 4, 1.9 %), dysphagia, and headache (*n* = 3, 1.4 % for each). ADRs were not recovered/resolved in nine subjects (4.2 %) at the end of the study [dysphagia was the ADR most frequently reported as not recovered/resolved (*n* = 3)]. The highest incidence of ADRs occurred after the first injection session of the EP, both in pre-treated (*n* = 23, 17.4 %) and treatment-naïve subjects (*n* = 30, 36.6 %). There was no cumulative effect of repeated injection sessions on the incidence of ADRs over the treatment period. Serious AEs were experienced by 11 subjects; none were considered to be related to study treatment.

Of 783 re-injections in the EP, 674 (86.1 %) were administered after an interval of ≥6 to ≤20 weeks; the mean (SD) injection interval was 14.0 (7.4) weeks and the median injection interval was 13.0 weeks. Of 191 subjects who received ≥2 injection sessions, the median injection interval was 6 to ≤10 weeks in 43 (22.5 %) subjects, >10 to ≤12 weeks in 47 (24.6 %) subjects, >12 to ≤14 weeks in 37 (19.4 %) subjects, and >14 weeks in 64 (33.5 %) subjects. In a post hoc analysis, there were no significant differences in the overall occurrence of AEs between these groups (*p* = 0.1117).

There were no clinically significant changes in hematology or clinical chemistry from the first injection session to the TTV. No pathological tendencies or deteriorations in neurological status were observed; most subjects had normal findings for all categories of the physical and neurological examinations at both the third injection session and the TTV.

### Neutralizing antibodies

Six subjects, who had all received botulinum toxin prior to the study, tested positive for neutralizing antibodies at screening for the main phase of the study and at the end of the extension phase of the study as assessed using the HDA (subsequent to a positive FIA).

## Discussion

This randomized, double-blind, repeated-dose, flexible-interval extension study, with a duration of up to 88 weeks (MP plus EP), shows that repeated injections of incobotulinumtoxinA, at total doses of 240 or 120 U, are efficacious and well tolerated in the treatment of CD. Improvements in mean TWSTRS scores were considered clinically relevant and comparable with results of published studies (Benecke et al. 2005; Brin et al. 2008; Comella et al. 2005, 2011; Truong et al. 2005).

IncobotulinumtoxinA was well tolerated with no unexpected safety risks following repeated injections. Dysphagia was the most common ADR, with incidences per injection interval comparable to rates described for other botulinum toxin preparations used to treat CD (Allergan, Inc 2011; Ipsen Biopharm Ltd 2009). Of note, in this study, unlike in previous studies of other botulinum toxins for CD, direct questioning using a 5-point dysphagia scale may have prompted a greater level of reporting by subjects. Incidences of ADRs did not increase with repeated injections, suggesting that there were no cumulative effects.

This is the first randomized trial in which flexible injection intervals were used in registration trials in the evaluation of repeated treatment of CD with botulinum toxin. Current US prescribing information for formulations of botulinum toxin type A suggests a minimum interval of 12 weeks between injection sessions, primarily due to concerns regarding safety and formation of neutralizing antibodies (Greene et al. 1994; Allergan Inc 2011; Merz Pharmaceuticals GmbH 2011; Ipsen Biopharm Ltd 2009). However, in this study of incobotulinumtoxinA, flexible injection intervals (minimum 6 weeks) were utilized at the discretion of the investigator depending on individual subject needs. This approach enabled individualized treatment for subjects who may derive greater benefit from more flexible dosing. In this study, 47.1 % of subjects with >2 incobotulinumtoxinA injections had a median injection interval ≤12 weeks and 22.5 % had a median injection interval ≤10 weeks. This is in line with the results of a multi-national survey of botulinum toxin injectors and subjects who were receiving onabotulinumtoxinA or abobotulinumtoxinA for the treatment of CD, which showed that >46 % of subjects would prefer shorter injection intervals (≤10 weeks) compared with those intervals actually received (Sethi et al. 2012). However, it has to be emphasized that the mean (SD) injection interval in this EP was 14.0 (7.4) weeks (median injection interval: 13.0 weeks). Importantly, the current study did not show significant differences in the incidence of AEs between subjects who received injection sessions at a median interval of 6 to ≤10 weeks and those treated less frequently, indicating that there are no additional safety concerns associated with shorter injection intervals when incobotulinumtoxinA is injected repeatedly according to subject needs with a flexible dosing period of up to 68 and a total duration of up to 88 weeks (up to six injections). For the 88-week duration of this study, there was no difference in the number of subjects testing positive for neutralizing antibodies in the HDA assay at the beginning of the main phase of the study versus the end of the EP. However, as the development of antibodies can occur with a longer treatment duration (Dressler 2002; Naumann et al. 2010) further studies with a larger number of subjects over a longer period are required to further evaluate the immunogenicity of repeated botulinum toxin treatments administered with flexible dosing intervals.

Neither the MP nor the EP was designed or powered to identify differences between the two dose groups and, accordingly, statistical analyses of treatment differences between the dose groups did not reach significance. To meet the requirements specified by regulatory authorities, subjects in this trial received randomized, fixed doses of incobotulinumtoxinA. Thus, total doses were not based on individual subjects’ medical needs and condition and did not take into consideration any previous botulinum toxin treatment outcomes subjects may have experienced, as would be standard practice in the treatment of CD outside a clinical trial setting. Re-randomization at the beginning of the EP also led to approximately two-thirds of subjects switching dose groups between the MP and the EP. As a result, subjects may have been over- or under-dosed, which may potentially have impacted on the frequency of AEs and/or withdrawals. Thus, it is inappropriate to draw definitive conclusions regarding differences between dose groups and purposely designed dose titration studies will be required to evaluate dosing for botulinum toxin treatment of CD. Nonetheless, similar to the results from the MP of this study, both doses of incobotulinumtoxinA demonstrated efficacy.

In summary, this randomized, double-blind extension study shows that repeated injections of incobotulinumtoxinA provide sustained efficacy for up to five treatment cycles when administered at total doses of 240 or 120 U using flexible dosing intervals according to subject need. Furthermore, incobotulinumtoxinA was well tolerated, and no unexpected safety risks emerged following repeated injections. No additional safety concerns were observed in subjects who received injection sessions at short intervals (6 to ≤10 or >10 to ≤12 weeks) compared with those who received injection sessions at longer intervals (>12 weeks).

## Study investigators

Richard Barbano (Rochester, NY, USA); Allison Brashear (Winston-Salem, NC, USA); Matthew Brodsky (Portland, OR, USA); Mahan Chehrenama (Alexandria, VA, USA); Cynthia Comella (Chicago, IL, USA); Paul Cullis (Detroit, MI, USA); Fabio Danisi (Kingston, NY, USA); Richard Dubinsky (Kansas City, KS, USA); Aaron Ellenbogen (Bingham Farms, MI, USA); Marian Evatt (Atlanta, GA, USA); Virgilio Gerald Evidente (Scottsdale, AZ, USA); Hubert Fernandez (Gainesville, FL, USA); Stephen Gollomp (Wynnewood, PA, USA); David Greeley (Spokane, WA, USA); Stephen Grill (Elkridge, MD, USA); Philip Hanna (Edison, NJ, USA); Robert Hauser (Tampa, FL, USA); Neal Hermanowicz (Irvine, CA, USA); Zhigao Huang (Jacksonville, FL, USA); Bahman Jabbari (New Haven, CT, USA); Joseph Jankovic (Houston, TX, USA); Un Jung Kang (Chicago, IL, USA); Mark LeDoux (Memphis, TN, USA); Kenneth Levin (Ridgewood, NJ, USA); Peter LeWitt (Southfield, MI, USA); Anthony Nicholas (Birmingham, AL, USA); Robert Rodnitzky (Iowa City, IA, USA); Alok Sahay (Cincinnati, OH, USA); Harvey Schwartz (Hollywood, FL, USA); Burton Scott (Durham, NC, USA); Kapil Sethi (Augusta, GA, USA); Dee Silver (La Jolla, CA, USA); Carlos Singer (Miami, FL, USA); Lynn Struck (Des Moines, IA, USA); William Sunter (Melbourne, FL, USA); Daniel Truong (Fountain Valley, CA, USA); Alberto Vasquez (St Petersburg, FL, USA); Maureen Wooten Watts (Dallas, TX, USA).
